# G6PD protects from oxidative damage and improves healthspan in mice

**DOI:** 10.1038/ncomms10894

**Published:** 2016-03-15

**Authors:** Sandrina Nóbrega-Pereira, Pablo J. Fernandez-Marcos, Thomas Brioche, Mari Carmen Gomez-Cabrera, Andrea Salvador-Pascual, Juana M. Flores, Jose Viña, Manuel Serrano

**Affiliations:** 1 Tumour Suppression Group, Spanish National Cancer Research Centre (CNIO), Madrid E28029, Spain; 2 Instituto de Medicina Molecular, Faculdade de Medicina, Universidade de Lisboa, 1649-028 Lisboa, Portugal; 3 Bioactive Products and Metabolic Syndrome Group, Madrid Institute of Advanced Studies (IMDEA) Food, Madrid E28049, Spain; 4 Université de Montpellier, INRA, UMR866, Dynamique Musculaire et Métabolisme, F-34060 Montpellier, France; 5 Department of Physiology, Faculty of Medicine, University of Valencia and Investigaciòn Hospital Clínico Universitario (INCLIVA), Valencia E46010, Spain; 6 Animal Surgery and Medicine Department, Faculty of Veterinary Sciences, Complutense University of Madrid, Madrid E28040, Spain

## Abstract

Reactive oxygen species (ROS) are constantly generated by cells and ROS-derived damage contributes to ageing. Protection against oxidative damage largely relies on the reductive power of NAPDH, whose levels are mostly determined by the enzyme glucose-6-phosphate dehydrogenase (G6PD). Here, we report a transgenic mouse model with moderate overexpression of human G6PD under its endogenous promoter. Importantly, G6PD-Tg mice have higher levels of NADPH, lower levels of ROS-derived damage, and better protection from ageing-associated functional decline, including extended median lifespan in females. The G6PD transgene has no effect on tumour development, even after combining with various tumour-prone genetic alterations. We conclude that a modest increase in G6PD activity is beneficial for healthspan through increased NADPH levels and protection from the deleterious effects of ROS.

## Abstract (web summary)

The enzyme G6PD generates the reductive metabolite NADPH, which has antioxidant effects, but has also been linked to tumour growth. Here the authors generate mice that modestly overexpress G6PD and report increased lifespan in females, and no negative effects on tumour formation in various genetic models.

## Graphical abstract

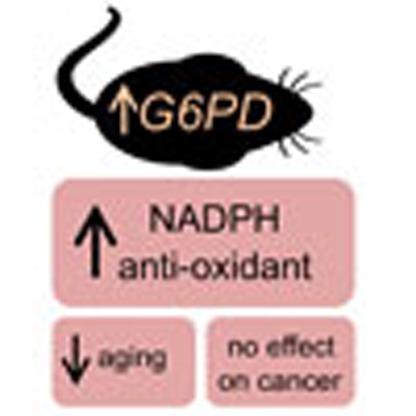

Reactive oxygen species (ROS) are generated as a result of normal intracellular metabolism and by the action of several external agents. ROS function as physiological signalling molecules that participate in the modulation of apoptosis, stress responses and proliferation. However, ROS can have a detrimental side by inflicting damage to macromolecules. Thus, organisms are endowed with antioxidant mechanisms that maintain ROS levels below a certain threshold under homoeostatic conditions^1^. During organismal ageing, regulation of ROS and oxidative repair become less efficient, thereby resulting in increased ROS production and accumulation of ROS-derived damage. The ‘free radical theory of ageing' argues that ROS-derived damage contributes to the functional decline of organ systems and predisposes to pathologies such as cancer, cardiovascular and neurodegenerative diseases^2^,^3^. In support of this, transgenic mice with increased expression of antioxidant enzymes are generally protected from a number of pathologies and in a few cases they present an increased lifespan^4^.

Thioredoxins, glutaredoxins and peroxiredoxins constitute a major antioxidant system that ultimately relies on the reductive power of NADPH^5^. NADPH also contributes to the maintenance of the active form of catalase, another important ROS-detoxifying enzyme^6^. Therefore, the homeostatic levels of NADPH are thought to determine the rate of ROS-derived damage^5^. Mammalian cells possess a few enzymes able to produce NADPH and, among them, glucose-6-phosphate dehydrogenase (G6PD) is considered the most important one^7^,^8^. G6PD catalyses the rate-limiting step in the pentose phosphate pathway (PPP), which provides nucleotide precursors for DNA replication, as well as NADPH reductive power for ROS detoxification and *de novo* lipid synthesis. The relevance of G6PD and the PPP in ROS detoxification is exemplified by the fact that mice deficient in G6PD have high levels of oxidative damage in the brain^9^. Also, human skin cells respond to acute oxidative stress by boosting their PPP-mediated NADPH production^10^. Finally, G6PD overexpression in *Drosophila melanogaster* protects against oxidative stress and can extend lifespan^11^.

Despite the central role of G6PD in antioxidant defence, nucleotide precursor synthesis and lipid synthesis, the consequences of G6PD overexpression in mammalian physiology have not been studied. Two points are of particular interest. First, G6PD may improve ROS detoxification and thereby reduce ROS-derived damage during ageing. Second, improved ROS detoxification and enhanced anabolism of nucleotides and lipids may favour tumoral growth. Here, we address these questions by generating transgenic mice with moderate ubiquitous overexpression of human G6PD under the control of its natural promoter.

## Results

### Generation of *G6PD* transgenic mice

**Figure 1:**
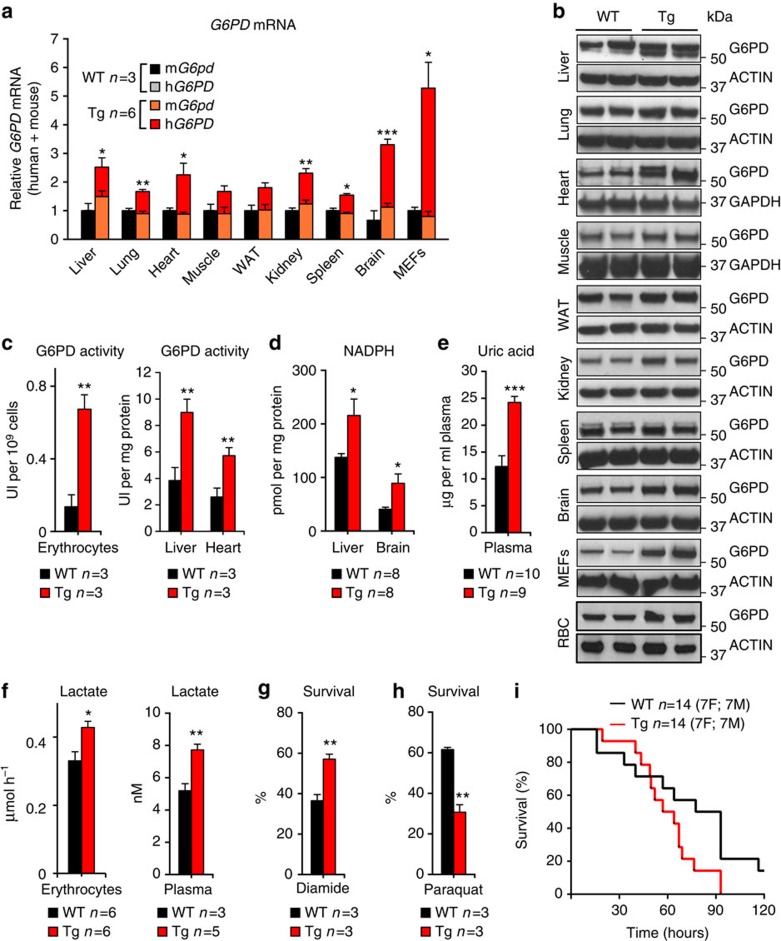
Characterization of the G6PD-Tg mice. (**a**) Relative *G6PD* mRNA of the indicated tissues from mice 10–12 weeks old, one male and two females for the WT group, one male (M) and five females (F) in the Tg group. (**b**) Levels of G6PD protein in the indicated tissues from 3 to 4-months-old mice and mouse embryo fibroblasts (MEFs) where each lane corresponds to one individual animal for the tissues (WAT: white adipose tissue; RBC: red blood cells), or an independent MEF preparation for the MEFs. The anti-G6PD antibody reacts with both the mouse and human G6PD protein. In some tissues (liver, heart), a second upper band was also detected. ACTIN or GAPDH were used as loading controls. (**c**) G6PD enzymatic activity in the indicated samples. (**d**) Levels of NADPH in the indicated tissues. (**e**) Uric acid concentration in plasma. (**f**) Lactate production in isolated erythrocytes (left panel) and total levels of lactate in plasma (right panel). (**g**,**h**) Percentage of cell survival in G6PD-Tg and WT primary MEFs upon treatment with the oxidative stress inducers diamide (**g**) and paraquat (**h**). (**i**) Survival curve of mice treated with a lethal dose of paraquat. Bars represent the mean±s.e.m. Statistic significance was assessed using the two-tailed Student's *t*-test for all experiments, with the exception of **i**, where the logrank test was applied: *P<0.05; **P<0.01; ***P<0.001.

For the generation of the G6PD transgenic mice, we used a large intact genomic fragment (20.1 Kb) comprising the entire human *G6PD* gene, including upstream and downstream regulatory sequences^12^. In this manner, we obtained a transgenic mouse line (G6PD-Tg) that expresses ∼2-fold levels of total *G6PD* mRNA (combined endogenous mouse mRNA and transgenic human mRNA) relative to wild-type (WT) littermates across all examined tissues and in mouse embryo fibroblasts (MEFs) (Fig. 1a). This was accompanied by a similar increase in protein levels (mouse and human proteins detected with the same antibody) (Fig. 1b; Supplementary Fig. 1).

The impact of the G6PD-Tg allele was measured first in erythrocytes, a cell type highly exposed to oxidative damage, and exclusively dependent on the PPP to obtain NADPH^13^. Importantly, erythrocytes from G6PD-Tg mice present a fivefold increase in G6PD enzymatic activity compared to WT littermates (Fig. 1c). Elevated G6PD enzymatic activity was also observed in liver and heart (Fig. 1c). In agreement with the role of G6PD in the maintenance of the NADPH pools, G6PD-Tg animals had significantly higher levels of NADPH in liver and brain (Fig. 1d). In addition to NADPH, the PPP generates nucleotides. In this regard, uric acid is the major end product of nucleotide metabolism and, therefore, uric acid levels can be used as a surrogate measurement of nucleotide metabolism. Interestingly, G6PD-Tg mice presented higher levels of plasma uric acid than their littermate controls (Fig. 1e). Finally, we also observed higher production of lactate in erythrocytes and increased total levels of lactate in plasma from G6PD-Tg mice, compared with WT controls (Fig. 1f). This increase in lactate is compatible with an elevated glycolytic flux, which could be due to the glycolytic intermediates generated by the PPP^14^,^15^. Together, these observations indicate that G6PD-Tg mice have constitutively increased levels of PPP activity and thereby higher levels of NADPH.

We wondered whether the higher NADPH levels observed in G6PD-Tg cells have a detectable impact in functional assays that depend on NADPH. For this, we first tested the survival of G6PD-Tg primary MEFs to diamide, a thiol-oxidant that oxidizes glutathione (GSH), thereby exhausting the NADPH reservoir^16^. In agreement with their elevated levels of G6PD expression, G6PD-Tg MEFs were more resistant to diamide compared with control WT MEFs (Fig. 1g). We also tested cell survival to paraquat, a highly toxic pro-drug whose activation by NADPH-cytochrome c reductase depends on the levels of NADPH^17^. Accordingly, G6PD-Tg MEFs were more sensitive to paraquat compared with WT MEFs (Fig. 1h). A similar trend was observed when mice were treated with paraquat, being G6PD-Tg mice more sensitive than WT mice to paraquat-induced lethality (Fig. 1i). In summary, two NADPH-dependent processes (resistance to diamide and sensitivity to paraquat) are enhanced by moderate G6PD overexpression. Altogether, we conclude that we have successfully generated a suitable tool to address the impact of G6PD overexpression and, thereby, higher NADPH levels in mouse physiology.

### Improved healthspan in G6PD-Tg mice

**Figure 2:**
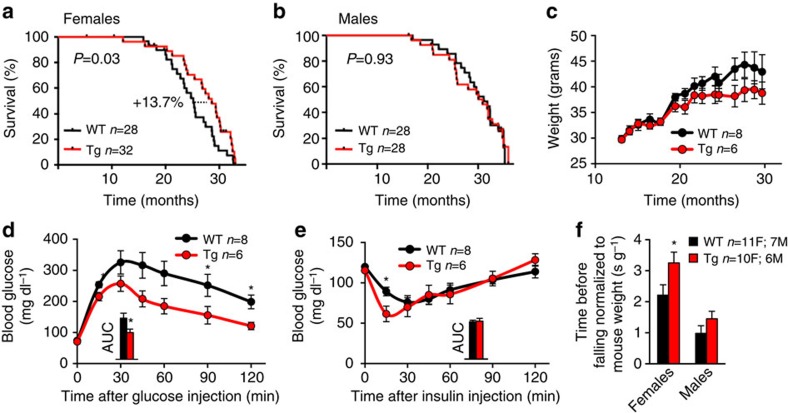
G6PD-Tg mice display improved healthspan. (**a**,**b**) Survival curves of female (**a**) and male (**b**) mice of the indicated genotypes. (**c**) Weight of males during the indicated time. (**d**,**e**) GTT (**d**) and ITT (**e**) on 1–year-old male mice. Inset, area under the curve (AUC). (**f**) Rotarod test on 1.5–2-year-old mice. Bars and dots represent average±s.e.m. Statistical significance was assessed using two-tailed Student's *t*-test with the exception of panels **a** and **b**, where logrank test was used: *P<0.05.

G6PD overexpression in flies extends longevity^11^ and based on this, we begun by studying the lifespan and ageing of G6PD-Tg mice. We followed cohorts of G6PD-Tg mice, together with their WT littermates, during their entire lifespan under standard chow diet. We observed a significant increase in the median lifespan of G6PD-Tg female mice (3.45 months or 13.7%), but not in G6PD-Tg males (Fig. 2a,b). No effects were observed in maximal lifespan in male or female G6PD-Tg mice. After detailed histopathological analyses of the deceased mice, we could not observe significant differences in the profile of pathologies developed by G6PD-Tg mice compared with WT control mice (Supplementary Fig. 2).

We also wondered whether G6PD overexpression could have an impact on organismal functional decline during ageing. For this, a homogeneous cohort of WT and G6PD-Tg mice of similar ages was used to perform phenotypic experiments. Ageing-associated obesity is a main risk factor for a number of diseases, most notably including diabetes^18^. Interestingly, old G6PD-Tg animals had a tendency towards lower body weight compared with their WT littermates (12% reduction in males and 9% reduction in females), although the differences were not statistically significant (Fig. 2c; Supplementary Fig. 3a). In addition, dual energy X-ray absorptiometry (DEXA) analysis revealed that old G6PD-Tg mice presented a normal lean mass and a slight reduction in fat mass, although the latter did not reach statistical significance (Supplementary Fig. 3b). Glucose intolerance and insulin resistance are associated to mouse ageing, and these processes are more pronounced in males compared to females. Interestingly, 1-year-old G6PD-Tg males were more glucose tolerant than their WT littermates, and presented improved insulin sensitivity at 15 min after insulin injection (Fig. 2d,e). At 6 months of age, G6PD-Tg males were equal to WT mice regarding glucose tolerance and insulin sensitivity (data not shown). In the case of females, glucose tolerance and insulin sensitivity were indistinguishable at one year of age between transgenic and WT genotypes (Supplementary Fig. 3c,d). We also performed a complete laboratory animal monitoring experiment on 1-year-old mice, measuring energy expenditure, food and water intake and locomotor activity. None of these parameters were changed between G6PD-Tg animals and their WT littermates (Supplementary Fig. 3e–h), indicating that the observed improvements in glucose tolerance and insulin sensitivity are not due to a significant alteration in energy expenditure, intake or activity. Finally, we tested neuromuscular fitness in aged mice using the rotarod test, and we observed a significant improvement in the performance of G6PD-Tg females and a non-significant trend for improvement in G6PD-Tg males at 1.5–2 years of age (Fig. 2f). In summary, G6PD overexpression in mice partially protects from ageing-associated functional decline, which depending on the sex manifests on different ageing-associated processes, including glucose tolerance, insulin sensitivity, neuromuscular fitness, and median lifespan.

### Cancer susceptibility is not affected in G6PD-Tg mice

**Figure 3:**
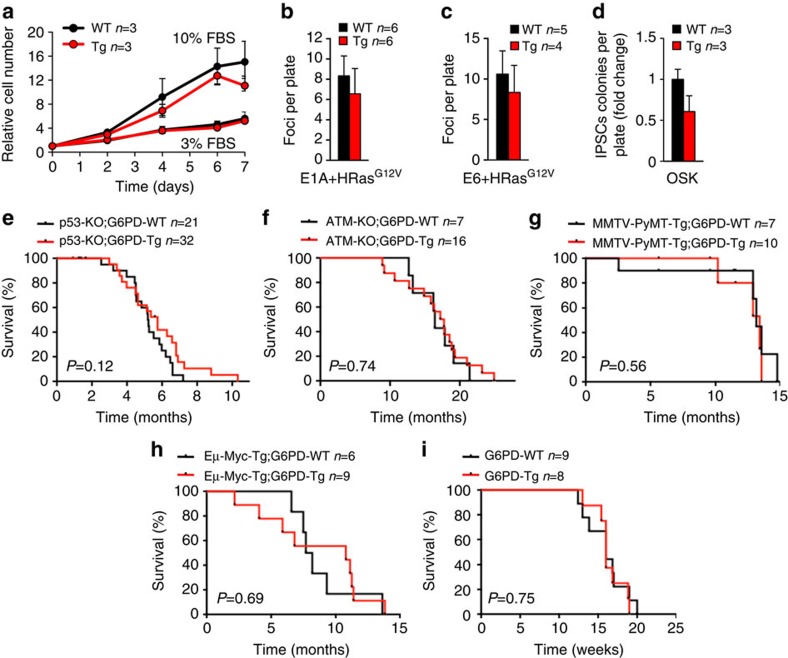
Cancer susceptibility is not affected in G6PD-Tg mice. (**a**) Growth curves of primary MEFs at the indicated serum concentrations. (**b**,**c**) Number of neoplastic foci in MEFs transduced with E1a/HRas^GV12^ (**b**) or with E6/HRas^G12V^ (**c**) after 2 weeks. (**d**) Fold change of reprogramming efficiency of primary MEFs after transduction with *Oct4*, *Sox2* and *Klf4*. (**e**–**i**) Survival curves of the indicated mouse lines and experiments. (**e**) p53-KO;G6PD-WT, *n*=21, 7 females (F); 14 males (M). p53-KO;G6PD-Tg, *n*=32, 12F, 20M. (**f**) ATM-KO;G6PD-WT, *n*=7, 3F, 4M; ATM-KO;G6PD-Tg, *n*=16, 6F, 10M. (**g**) MMTV-PyMT-Tg;G6PD-WT, *n*=7F; MMTV-PyMT-Tg;G6PD-Tg, *n*=10F. (**h**) Eμ-Myc-Tg;G6PD-WT, *n*=6, 3F, 3M; Eμ-Myc-Tg;G6PD-Tg, *n*=9, 4F, 5M. (**i**) Survival curve of mice injected intramuscularly with 3MC, G6PD-WT *n*=9, 1F, 8M; G6PD-Tg *n*=8M. Bars represent mean±s.e.m. Statistical significance was determined by the two-tailed Student's *t*-test for **a**–**d**, and by the logrank test for **e**–**i**. None of the differences was statistically significant.

G6PD has been proposed to have pro-oncogenic properties based on its overexpression in several tumour types and studies with *in vitro* cultured cells^19^,^20^,^21^,^22^. In particular, overexpression of G6PD in murine fibroblasts under the actin promoter is able to increase their proliferation rate and may induce oncogenic transformation^22^. We wondered if this could also be the case in our G6PD-Tg MEFs, which overexpress G6PD under its natural promoter. G6PD-Tg MEFs proliferated at the same rate than their WT controls at different concentrations of serum (Fig. 3a), and formed the same number of neoplastic foci after co-transduction of oncogenes E1A and HRas^G12V^ (Fig. 3b) or E6 and HRas^G12V^ (Fig. 3c). The process of reprogramming of differentiated cells into induced pluripotent stem cells (iPSC) shares some similarities with neoplastic transformation and it can provide a readout of transformability and oncogenic potential^23^. Interestingly, G6PD-Tg MEFs formed the same number of iPSC colonies than WT MEFs upon reprogramming with *Oct4, Klf4* and *Sox2* (Fig. 3d).

At the organismal level, we did not detect any significant increase in the incidence of spontaneous cancers in G6PD-Tg animals (Supplementary Fig. 2). To further investigate *in vivo* the possible oncogenic activity of G6PD, we combined the G6PD-Tg allele with different pro-oncogenic backgrounds. In particular, *p53*-null (p53-KO), which develop mostly sarcomas and T-cell lymphomas^24^; *Atm*-null (ATM-KO), which develop T-cell lymphomas^25^; MMTV-PyMT (polyoma middle-T oncoprotein under the mouse mammary tumor virus promoter), mammary carcinomas^26^; and Eμ-*Myc*, B-cell lymphomas^27^. Notably, survival of all these tumour-prone mouse lines was indistinguishable regardless of the presence of the G6PD-Tg allele (Fig. 3e–h). Finally, we subjected G6PD-Tg mice and their WT littermates to 3-methyl-cholantrene (3MC), a potent carcinogen that induces fibrosarcomas in a p53 and p19^Arf^-dependent fashion^28^,^29^, and G6PD-Tg mice developed tumors with the same kinetics as WT controls (Fig. 3i).

All together, we conclude that moderate overexpression of G6PD under its natural promoter does not result in increased tumorigenesis.

### Lower ROS-derived damage in aged G6PD-Tg mice

**Figure 4:**
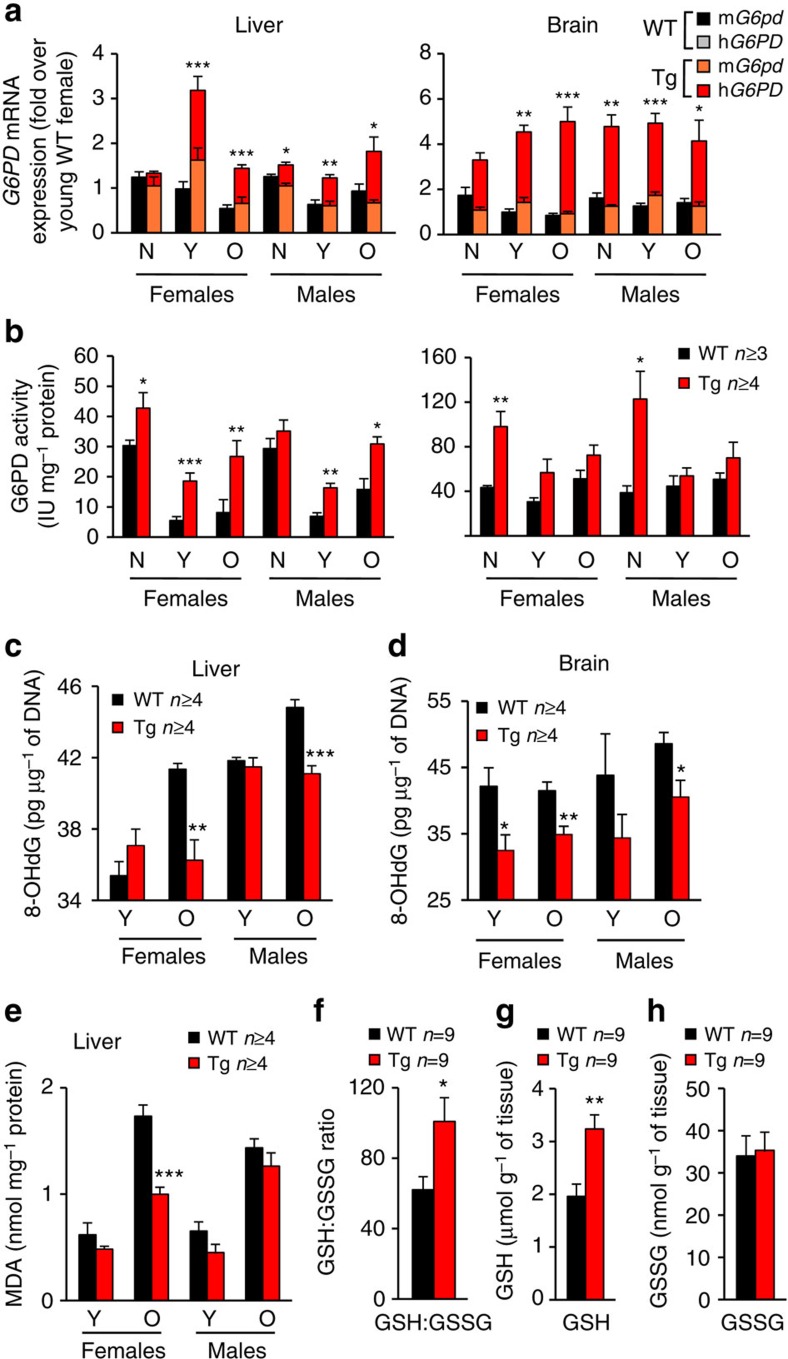
Overexpression of G6PD *in vivo* protects from oxidative damage. (**a**,**b**) G6PD mRNA expression levels (**a**) and G6PD activity (**b**) in liver (left panels) and brain (right panels) of neonates, young (10–35 weeks) and old (125–143 weeks) female or male mice. For RNA measures: neonates, *n*=5 for all groups. Liver: young WT females, *n*=11; young Tg females, *n*=13; old WT females, *n*=22; old Tg females, *n*=22; young WT males, *n*=12; young Tg males, *n*=13; old WT males, *n*=13; old Tg males, *n*=11. Brain: young WT females, *n*=3; young Tg females, *n*=6; old WT females, *n*=15; old Tg females, *n*=15; young WT males, *n*=4; young Tg males, *n*=6; old WT males, *n*=6; old Tg males, *n*=5. For G6PD activity: neonates, *n*=4 for all groups. Liver: young WT females, *n*=8; young Tg females, *n*=8; old WT females, *n*=4; old Tg females, *n*=5; young WT males, *n*=4; young Tg males, *n*=4; old WT males, *n*=5; old Tg males, *n*=10. Brain: young WT females, *n*=3; young Tg females, *n*=4; old WT females, *n*=4; old Tg females, *n*=5; young WT males, *n*=4; young Tg males, *n*=4; old WT males, *n*=5; old Tg males, *n*=4. (**c**,**d**) DNA oxidation (8-OHdG adducts) in liver (**c**) or brain (**d**). (**e**) Lipid oxidation (MDA adducts) in liver. (**f**–**h**) GSH:GSSG ratio (**f**) and GSH (**g**) or GSSG (**h**) concentrations were assayed in liver samples from 2-year-old female mice. Bars represent mean±s.e.m. Statistical significance was assayed using the two-tailed Student's *t*-test: *P<0.05; **P<0.01; ***P<0.001.

ROS-derived damage increases continuously with ageing^30^,^31^ and some antioxidant manipulations delay ageing^4^. Based on this, we hypothesized that increased G6PD expression and activity could lead to decreased ROS-derived damage. To test this, we first analysed the expression and activity of G6PD in liver, brain and muscle from neonates (0–2 days), young (10–35 weeks) and old (125–143 weeks) G6PD-Tg and WT mice. Expression and activity of G6PD were increased in the liver and brain of young and old G6PD-Tg mice (Fig. 4a,b). Higher G6PD activity was also observed in the muscle from young and old G6PD-Tg mice (Supplementary Fig. 4a). Of note, G6PD-Tg expression was slightly higher in females compared to males, which could be due to the known positive regulation of *G6PD* expression by oestrogens^32^,^33^. We wondered if G6PD overexpression could have an impact on the expression of antioxidant genes with ageing. We observed a modest increase in the mRNA levels of some antioxidant genes (*Cat*, *Sod1* and *Txnrd1*) in young female mice (Supplementary Fig. 4b). However, at old age, all the examined antioxidant genes showed a global reduction in expression relative to young mice and no differences were observed between WT and G6PD-Tg mice regardless of their sex (Supplementary Fig. 4b). The decrease in expression of antioxidant enzymes with age had been previously reported in rodents^30^ and humans^31^. Finally, the total protein levels of several redox catalysts, such as thioredoxins, glutaredoxins and peroxiredoxins, were not altered in young or old G6PD-Tg mice (Supplementary Fig. 4c).

To assess the impact of lifelong overexpression of *G6PD* on ROS-derived damage, we measured the levels of macromolecular oxidative damage in young and old mice. Importantly, old G6PD-Tg male and female mice showed diminished accumulation of DNA oxidation (measured as 8-hydroxyguanosine or 8-OHdG) in liver and brain (Fig. 4c,d). Old females also showed reduced lipid oxidation (measured as malondialdehyde or MDA) in the liver (Fig. 4e; Supplementary Fig. 5a), but not in the brain (Supplementary Fig. 5b). No changes were observed in protein oxidation (measured as carbonylated proteins) in the liver of old male or female G6PD-Tg mice (Supplementary Fig. 5c,d). Of note, old G6PD-Tg males, but not females, presented a small but significant increase in brain protein carbonylation (Supplementary Fig. 5e,f). In accordance with these findings, and further supporting the role of G6PD in NADPH-dependent ROS detoxification, liver from 2-year-old G6PD-Tg female mice presented an elevated reduced versus oxidized gluthatione ratio (GSH:GSSG) ratio (Fig. 4f), which is indicative of improved ROS detoxification^34^. This increased GSH:GSSG ratio was due to an elevation in reduced GSH (Fig. 4g), without a detectable change in oxidized glutathione (GSSG) (Fig. 4h). Since the conversion of GSSG to GSH (by glutathione reductase) requires NADPH, our results again indicate that G6PD-Tg animals, by having increased levels of NADPH, possess a more robust degree of ROS detoxification, and thus lower accumulation of ROS-derived damage at old age.

## Discussion

Ageing is a complex process driven by multiple sources of damage, both internal and external. Genetic studies in animal models have uncovered genes that are able to affect the ageing process, but their discovery and characterization still remains challenging, particularly in mammals. Our work uncovers a novel genetic manipulation that improves some aspects of ageing in mammals.

G6PD is the rate-limiting enzyme for the PPP, which plays two important roles in cell physiology: synthesis of nucleotide precursors, and production of NADPH, which in turn is the main determinant of the antioxidant capacity of cells. Here, we have generated transgenic mice with modest (∼2-fold) overexpression of G6PD and we show *in vivo* that this reflects in higher levels of NAPDH and in higher production of nucleotide precursors (measured as uric acid in the blood).

Phenotypically, analyses of G6PD-Tg mice at different ages have revealed that these mice are partially protected from some ageing-associated processes. In particular, old G6PD-Tg mice presented improved glucose tolerance and insulin sensitivity in males, and improved rotarod function (an indicator of neuromuscular fitness) and increased median lifespan in females. These results are in line with previous overexpression mouse models of NADPH-dependent ROS-detoxifying enzymes, some of which present better glucose tolerance or insulin sensitivity (PRX3-Tg or PRX4-Tg mice), neuroprotection (GPX1-Tg or GRX2-Tg mice), protection from cardiovascular damage (GPX4-Tg, TRX1-Tg, GRX1-Tg and GRX2-Tg mice), or extended median lifespan without increased maximum lifespan (TRX1-Tg mice)^4^. The observed improvement in healthspan in G6PD-Tg mice also coincides with observations in flies, where G6PD overexpression increased protection from ROS and maximum lifespan^11^.

Of interest, G6PD overexpression has been found in a number of human cancers, and G6PD has been proposed to be pro-oncogenic^19^,^20^,^21^,^22^. However, our *in vitro* and *in vivo* data indicate that moderate overexpression of G6PD does not affect tumour onset in a WT background, nor in many other tumour-prone backgrounds (*p53*-null, *Atm*-null, MMTV-PyMT or Eμ-*Myc*). We conclude that G6PD overexpression in our G6PD-Tg mice has no impact on tumorigenesis.

Given the importance of NADPH in antioxidant defences, we also analysed molecular markers of oxidative damage in aged mice. Importantly, liver and brain from old G6PD-Tg mice, male and female, presented lower levels of oxidized DNA. In contrast to this, G6PD-deficient mice have higher levels of oxidized DNA (ref. 9). G6PD-Tg females also presented decreased lipid oxidation in the liver. This protection against oxidative molecular damage was associated to a higher ratio between reduced and oxidized glutathione in the liver of old mice.

Together, these observations support a mechanistic link between increased G6PD activity, elevated NADPH and redox potential, and improved antioxidant protection, and this could explain the beneficial health effects observed in old G6PD-Tg mice.

## Methods

### Generation of transgenic mice

Transgenic mice were generated at the Spanish National Cancer Research Center (CNIO) at the Transgenic Mice core facility and are housed at the specific pathogen free barrier areas of the CNIO Animal House core facility. G6PD-Tg mouse line was generated using a 20 Kb human genomic DNA construct containing the entire *G6PD* gene, including 2.5 Kb of upstream flanking sequence and 2.0 Kb of downstream flanking sequence^12^. This *G6PD* sequence was isolated from the pBluescript vector by NotI digestion and a 0.5 to 1 ng μl^−1^ DNA solution was injected into the pronuclei of F1 hybrids (C57BL/6J × CBA) fertilized oocytes using standard microinjection procedures. The resulting offspring was analysed for the presence of the transgene by PCR reaction using primers specific for the human *G6PD* gene (Forward: 5′- AAGAAGCAGACTGGAGGAGAAG -3′ and Reverse: 5′- CAGGTTGTCACTCTCAGAACAGA -3′) and that do not hybridize to the homologous mouse *G6pd* gene. One founder capable of transmitting the transgene to the progeny and that overexpressed G6PD was identified (+/+;tg), abbreviated here as G6PD-Tg. The founder G6PD-Tg mouse was backcrossed for three generations with pure C57BL6 mice; in this manner, all of the mice used in this study share a genetic background that is 93.75% C57BL6. All the C57BL6 mice were purchased from Harlan Laboratories and correspond to the sub-strain C57BL6/J-OlaHsd.

### Animal experimentation

Animal experimentation at the CNIO, Madrid, was performed according to protocols approved by the CNIO-ISCIII Ethics Committee for Research and Animal Welfare (CEIyBA).

DEXA on 13 females (5 WT and 8 G6PD-Tg) and 14 males (8 WT and 6 G6PD-Tg) of 1 year of age was performed with isofluorane-anesthetized mice in a Lunar PixiMUS (GEHC) apparatus.

GTT was performed on O/N fasted animals, 11 females (5 WT and 6 G6PD-Tg) and 14 males (8 WT and 6 G6PD-Tg) of 1 year of age. Mice were injected IP with 2 g kg^−1^ dextrose (Sigma) dissolved in sterile saline, and blood glucose levels were measured with Glucocard strips (A. Menarini Diagnosis) at different time points, while animals were allowed to drink freely.

ITT was performed in non-fasted animals, 13 females (5 WT and 8 G6PD-Tg) and 14 males (8 WT and 6 G6PD-Tg) of 1 year of age. Mice were injected with 0.75 U kg^−1^ insulin (Humumil, Lilly), and blood levels were measured with Glucocard strips (A. Menarini Diagnosis) at different time points, while animals were allowed to drink freely.

Rotarod test was performed using the Rotarod apparatus (Panlab). Specifically, 21 females (11 WT and 10 G6PD-Tg) of 2 years of age and 13 males (7 WT and 6 G6PD-Tg) of 1.5 years of age were trained with 3 rounds of rotarod tests for 3 days, and at the 4th day, data from 3 successive experiments were recorded. Final data is the average of the 3 experiments performed the 4th day.

Complete laboratory animal monitoring system was performed on 13 females (5 WT and 8 G6PD-Tg) and 14 males (8 WT and 6 G6PD-Tg) of 1 year of age using the Oxylet apparatus (Panlab-Harvard Apparatus). Briefly, acclimatization of mice to the measurement cages was performed 3 days before data recording. Room temperature was constantly kept at 21 °C, while running light/dark cycles of 12 h. Respiratory quotient (RQ) was calculated as RQ=VCO_2_/VO_2_ from volumes of consumed O_2_ (VO_2_) and eliminated CO_2_ (VCO_2_) recorded every 20 min. We calculated energy expenditure (EE) as EE=(3.815+(1.232 × RQ)) × VO_2_ × 1.44). Moreover, we recorded food and water intake and locomotor activity in time intervals of 20 min during the whole measurement period.

Resistance to paraquat: 14 female (7 WT and 7 G6PD-Tg) and 14 male (7 WT and 7 G6PD-Tg) mice of 4 to 6 months of age were intraperitoneally injected with a lethal dose of paraquat (60 mg kg^−1^) and their survival fraction was scored every 12 h. Experiment was terminated 5 days (120 h) post-paraquat inoculation.

For 3-methyl-cholanthrene (3MC) carcinogenesis, 1 WT female and 16 male (8 WT and 8 G6PD-Tg) mice of 3 months of age received a single intramuscular injection at one of the rear legs of a 100 μl solution containing 3MC (Sigma), at a concentration of 100 μg μl^−1^ and dissolved in sesame oil (Sigma) and the time point at which tumors reach 1.5 cm maximum diameter was scored. For cancer susceptibility, G6PD-Tg animals with a genetic background higher than 96.875% C57BL6 (F4) were crossed with C57BL6 pro-oncogenic mouse strains and cohorts of animals were followed and sacrificed when showing overt signs of morbidity according to the Humane End Point criteria. Specifically, G6PD animals were crossed to *p53*-null (−/−) animals to generate p53-KO;G6PD-WT (*n*=21, 7 females (F) and 14 males (M)) and p53-KO;G6PD-Tg (*n*=32, 12F and 20M) mouse lines; to *Atm* heterozygous (+/−) animals to generate ATM-KO;G6PD-WT(*n*=7, 3F and 4M) and ATM-KO;G6PD-Tg (*n*=16, 6F and 10M) mouse lines; to MMTV-PyMT-Tg hemizygous transgenic animals to generate MMTV-PyMT-Tg;G6PD-WT (*n*=7F) and MMTV-PyMT-Tg;G6PD-Tg (*n*=10F) mouse lines and to Eμ-Myc-Tg hemizygous transgenic animals to generate Eμ-myc-Tg;G6PD-WT (*n*=6, 3F and 3M) and Eμ-myc-Tg;G6PD-Tg (*n*=9, 4F and 5M) mouse lines.

### Histopathology

Mice were sacrificed when evident signs of morbidity were observed. A comprehensive collection of tissues were fixed in 10% buffered formalin O/N at room temperature, included in paraffin and sections were stained with hematoxilin–eosin. All slides were analysed by an expert pathologist in a blind manner with regard to the genotype of the mice.

### Western blot

Whole-cell extracts were prepared using RIPA buffer containing proteinase inhibitors, resolved using NuPAGE 4–12% gradient Bis-Tris gels, transferred to nitrocellulose and hybridized using antibodies against G6PD (1:1,000; Abcam, ab993), ACTIN (1:10,000; Sigma, A5441), GAPDH (1:10,000; Sigma, G8795), α-Tubulin (1:1,000; Sta Cruz, sc-8035), Thioredoxin-1 (1:1000; Cell Signaling, 2429), Thioredoxin-2 (1:1000; Proteintech, 13089-1-AP), Glutaredoxin-1 (1:1000; Abcam, Cab55059), Glutaredoxin-2 (1:1000; Abcam, ab85267), Peroxiredoxin-6 polyclonal antibody (1:1000; Abcam ab59543). HRP-coupled secondary antibodies (1:2,000) were from Dako Denmark A/S. Uncropped images of all the immunoblots can be found in Supplementary Figs 6 and 7.

### RNA analyses

Total RNA from tissues or cells was extracted using TRIZOL (Life Technologies) followed by RNA cleanup (Qiagen, 74104) and DNase I treatment (Qiagen) in column. Samples were reverse transcribed using random priming and Superscript Reverse Transcriptase (Life Technologies), according to the manufacturer's instructions. Quantitative real-time PCR was performed using DNA master SYBR Green I mix (Applied Biosystems) in an ABI PRISM 7700 thermocycler. Quantifications were made applying the ΔCt method (ΔCt=(Ct of gene of interest−Ct of housekeeping)). The G6PD fold expression corresponds to the sum of the 2^(−ΔCt)^ for the mouse G6PD and human G6PD primers. The housekeeping gene used for input normalization was β-actin. Primer sequences are described in Supplementary Table 1.

### Cell culture experiments

For viability assays, MEFs were treated with 0.6 mM diamide for 4 h or 0.4 mM paraquat for 24 h. Cells were then stained with cresyl violet (Sigma), and cellular viability was determined by measuring the absorbance at 595 nm.

For MEFs growth curves, 2500 cells were plated in 96-well plates and in different concentrations of serum (3 and 10%). At 0 (24 h after plating), 2, 4, 6 and 7 days, cells were stained with cresyl violet and cell concentration was determined by measuring absorbance at 595 nm.

For transformation assays, MEFs were transduced with retroviral vectors carrying (a) the oncogenes E1A and HRas^G12V^ in the same policistronic construct, or (b) two lentiviral vectors carrying the oncogenes E6 and HRas^G12V^, respectively. After transduction, 5,000 cells (E1A-HRasG12V) or 20,000 cells (E6+HRasG12V) were plated in P100 plates, and cultured in DMEM+10% fetal bovine serum and penicillin–streptomycin for 2 weeks. Cells were then fixed with formaldehyde at a final concentration of 6%, stained with Giemsa (Sigma), and foci were counted.

Reprogramming of primary (passage 2–4) MEFs was performed as previously described^35^. In brief, retroviral supernatants were produced in HEK-293T cells (purchased from Invitrogen) (5 × 10^6^ cells per 100-mm-diameter dish) transfected with the ecotropic packaging plasmid pCL-Eco (4 μg) together with one of the following retroviral constructs (4 μg): pMXs-Klf4, pMXs-Sox2 or pMXs-Oct4. Transfections were performed using Fugene-6 transfection reagent (Roche) according to the manufacturer's protocol. Two days later, retroviral supernatants (10 ml) were collected serially during the subsequent 48 h, at 12-h intervals, each time adding fresh medium to the cells (10 ml). The recipient MEFs had been seeded the previous day (2.5 × 10^5^ cells per 100-mm-diameter dish) and received 1.5 ml of each of the corresponding retroviral supernatants. This procedure was repeated every 12 h for 2 days (a total of four additions). After infection was completed, medium was replaced by complete KnockOut Serum Replacement (KSR) medium, composed of DMEM (25 mM glucose) supplemented with serum replacement (KSR, 15%, Invitrogen), leukemia inhibitory factor (LIF) (1,000 U ml^−1^), non-essential amino acids, glutamax and β-mercaptoethanol. Cultures were maintained in the absence of drug selection with daily medium changes. From days 10 to 12 colonies with ES-cell-like morphology became visible and were scored after alkaline phosphatase staining.

### Biochemical measurements

G6PD activity was determined as described elsewhere^36^. Briefly, 1,000 μl of glucose-6-phosphate (10 mM final concentration) in potassium phosphate buffer was added to a cuvette. Then 400 μl of liver or brain homogenate were introduced. The reaction was initiated by 400 μl of NADP (0.9 mM final concentration) in buffer. The mixture was inverted and the absorbance read over 3 min at 340 nm using a spectrophotometer. Protein concentrations were determined by Bradford's method with bovine serum albumin as standard.

NADPH was determined from tissue lysates using the NADP^+^/NADPH quantification kit (BioVision), following manufacturer's instructions.

Plasma uric acid concentration assessment was carried into a Randox Daytona automate using a commercial kit (URIC ACID Liquid Mono Reagent, COLORIMETRIC RX DaytonaTM, Randox, France) according to the manufacturer's protocol.

For plasma lactate measurements, blood sample from the abdominal aorta were collected in Lithium heparin tubes (microvette, SARSTEDT) and centrifuged at 2,000*g* during 5 min at room temperature according to the manufacturer's instructions. Plasma was then collected and stored at −80 °C for further analysis. About 50 μl of plasma was added to a cuvette containing a hydrazine/glycine buffer with NAD^+^ and lactate dehydrogenase and incubated with for 30 min at 37 °C. NADH formation is measured by the increase in extinction at 340 nM and corrected by subtracting the value obtained for the reagent blank. Values are expressed in mM.

Lactate production from erythrocytes was measured according to previous methods^37^. Briefly, blood sample from the abdominal aorta were collected in Lithium heparin tubes (microvette, SARSTEDT) and centrifuged at 1,200*g* during 5 min at room temperature. Plasma and buffy coat were removed and erythrocytes were washed 3 times with Krebs–Ringer phosphate buffer containing 0.2 mM glucose and pH 7.4. Then, erythrocytes were resuspended in the same buffer to obtain a 40% haematocrit with a final glucose concentration of 5 mM and incubated during 180 min at 37 °C with gentle agitation. The reaction was stopped by addition of 0.6 N of HClO_4_. Denatured proteins were sedimented by centrifugation for 5 min at 10,000*g*, and the supernatants were used for the lactate assay using the method previously described. Data are expressed in μmol h^−1^.

Oxidative DNA damage was measured by 8-hydroxy-2′-deoxyguanosine (8-OHdG) as previously described^38^. A commercially available enzyme linked immunoassay (OxiSelect Oxidative DNA Damage ELISA Kit, Cell Biolabs) was used to measure oxidized DNA in isolated liver and brain DNA samples. DNA was extracted from liver and brain via the High Pure PCR Template Preparation Kit (Roche, GmbH, Germany) according to the manufacturer's protocol. DNA was only used if it had a minimum 260:280 ratio of 1.8. The assay was performed following the manufacturer's directions. Briefly, 50 μl of DNA or 8-OHdG standards were first added to an 8-OHdG/BSA (bovine serum albumin) conjugate preabsorbed microplate. After a brief incubation, an anti-8-OHdG monoclonal antibody was added, followed by an HRP conjugated secondary antibody. The 8-OHdG content in unknown samples is determined by comparison with predetermined 8-OHdG standard curve. Samples were normalized to the DNA concentration measured via a plate spectrophotometer for nucleic acids (Eppendorf, BioPhotometer). All analyses were done in duplicate. Data are expressed in pg μg^−1^ of DNA.

Lipids peroxidation determination as malondialdehyde (MDA) in brain and liver were performed as described elsewhere^39^. Briefly, this method is based on the hydrolysis of lipid peroxides and subsequent formation of the adduct thiobarbituric acid (TBA) and MDA (TBA–MDA2). This adduct is detected by reverse phase HPLC and quantified at 532 nm (Ultimate 3000 Dionek). The chromatographic technique was performed in isocratic mobile phase being a mixture of 50 mM KH_2_PO_4_ (pH 6.8) and acetonitrile (70:30)

Oxidative modification of total proteins in liver and brain were assessed by immunoblot detection of protein carbonyl groups using the ‘OxyBlot' protein oxidation kit (Millipore, MA, USA)^40^. About 20 μg of total protein was loaded onto gels and electrophoretically separated. Antibody anti-dinitrophenylhydrazone was purchased from Intergen Company (Purchase, NY,USA). Total protein carbonyls were quantified with the OxyBlot kit by densitometry of the blotting and of the Ponceau red staining, followed by finding the ratio between the total density in the oxyblot and the total density in the Ponceau red staining. Specific proteins were visualized by using the enhanced chemiluminescence procedure as specified by the manufacturer (Amersham). Autoradiographic signals were assessed using a BioRad scanning densitometer.

Determination of GSH and GSSG was carried out using the high-performance liquid chromatography method with ultraviolet–visible detection, which we developed to measure GSSG in the presence of a large excess of GSH^41^. The essence of this method consists of minimizing GSH oxidation, which otherwise would result in a large increase in GSSG. To measure GSH, liver homogenates (0.5 ml) were treated at 4 °C with 0.5 ml ice-cold perchloric acid (12%) containing 2 mM bathophenanthroline disulfonic acid (BPDS; Sigma Chemical Co.). For GSSG determination, samples (0.5 ml) were treated, at 4 °C, with 0.5 ml ice-cold perchloric acid (12%) containing 40 mM *N*-ethylmaleimide (Sigma Chemical Co., St Louis, MO, USA), to prevent GSH oxidation, and 2 mM BPDS. Samples were then centrifuged at 15,000*g* for 15 min, at 4 °C, and the acidic supernatants were used for total glutathione and GSSG measurements.

### Statistical methods

Statistical tests and significances are specified at every figure legend. Sample collection was always performed using biological replicates (individual clones in *in vitro* experiments, different mouse individuals in *in vivo* experiments). Samples (cells or mice) were allocated to their experimental groups according to their predetermined type (cell type or mouse genotype) and therefore there was no randomization. Except for the pathological analyses, investigators were not blinded to the experimental groups (cell types or mouse genotypes). Normal distribution of the samples groups was assessed by the Shapiro–Wilk test. Equal variances of the samples groups were assessed by the *F*-test.

## Authors contributions

S.N.-P. and P.J.F.-M. performed most of the experiments, contributed to experimental design, data analysis, discussion and writing; M.C.G.-C., T.B. and A.S.-P. contributed to experimental work and design, discussion and writing; J.M.F. performed the histopathological analysis; J.V. contributed to experimental design, discussion and writing; M.S. designed and supervised the study, secured funding, analysed the data, and wrote the manuscript. All authors discussed the results and commented on the manuscript.

## Additional information

**How to cite this article:** Nóbrega-Pereira, S. *et al*. G6PD protects from oxidative damage and improves healthspan in mice. *Nat. Commun.* 7:10894 doi: 10.1038/ncomms10894 (2016).

## Supplementary Material

Supplementary Information Supplementary Figures 1-6 and Supplementary Table 1
