# Supplementary material for: G6PD protects from oxidative damage and improves healthspan in mice
Source: Nat Commun. 2016 Mar 15;7:10894. doi: 10.1038/ncomms10894 (PMC4796314; doi:10.1038/ncomms10894)
Supplement: Supplementary Information — Supplementary Figures 1-6 and Supplementary Table 1 [file ncomms10894-s1.pdf]

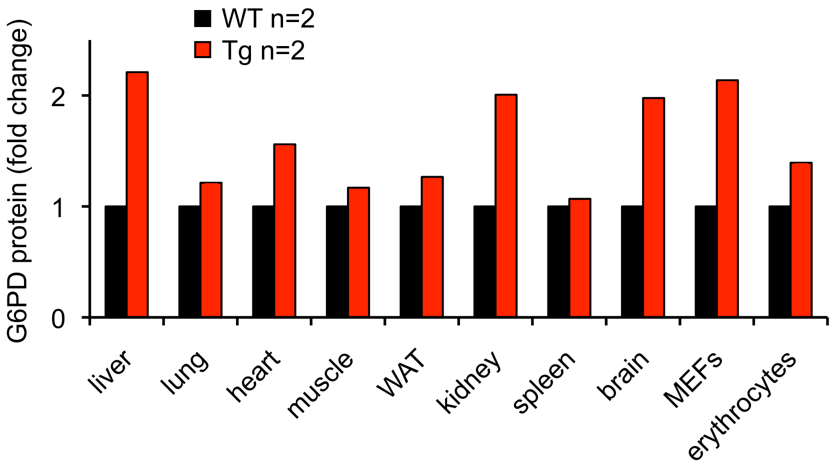

Supplementary Figure 1. **G6PD protein expression in different tissues.** Quantification of the WB presented in Figure 1b. Values correspond to the average of two values (n=2).

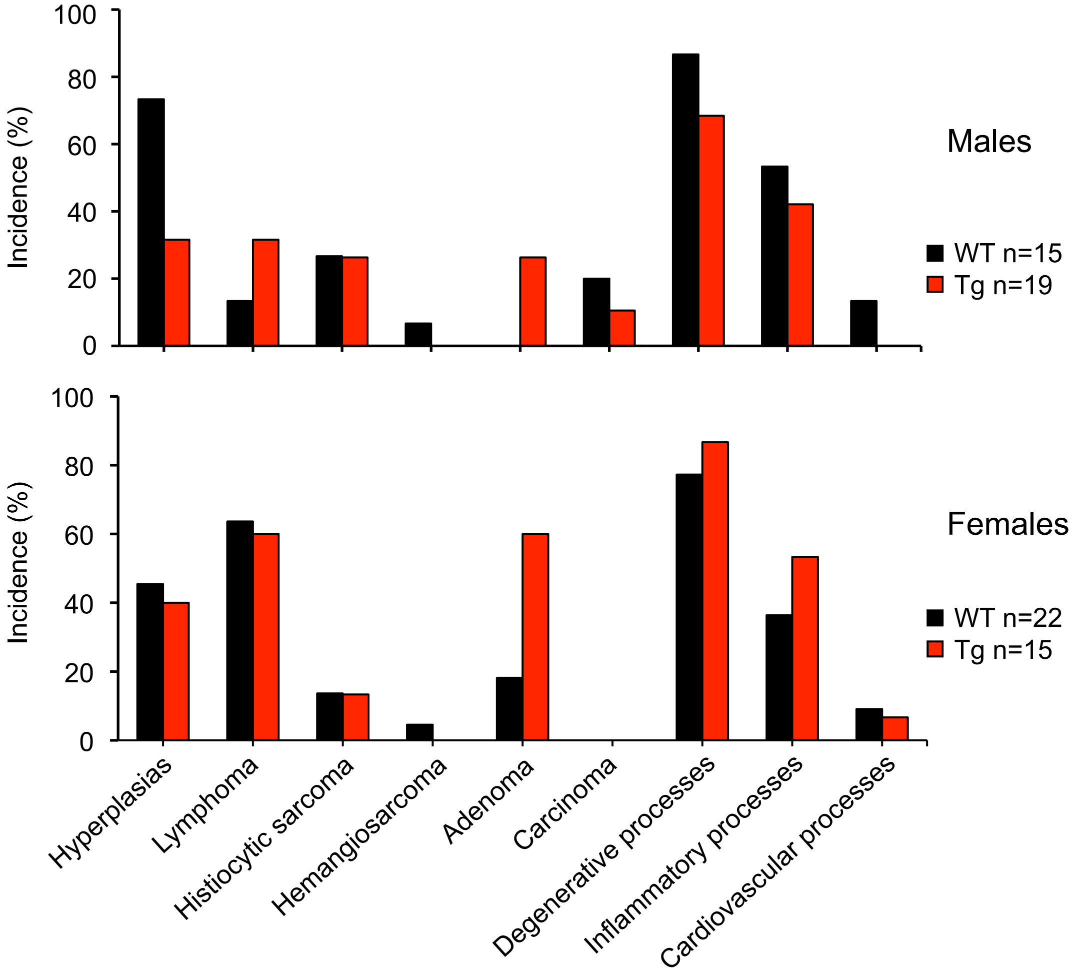

Supplementary Figure 2. Histopathological analysis performed on mice used to generate Figures 2a and 2b. Fisher's exact test was performed to assess differences in incidence between genotypes. No significant differences were observed.

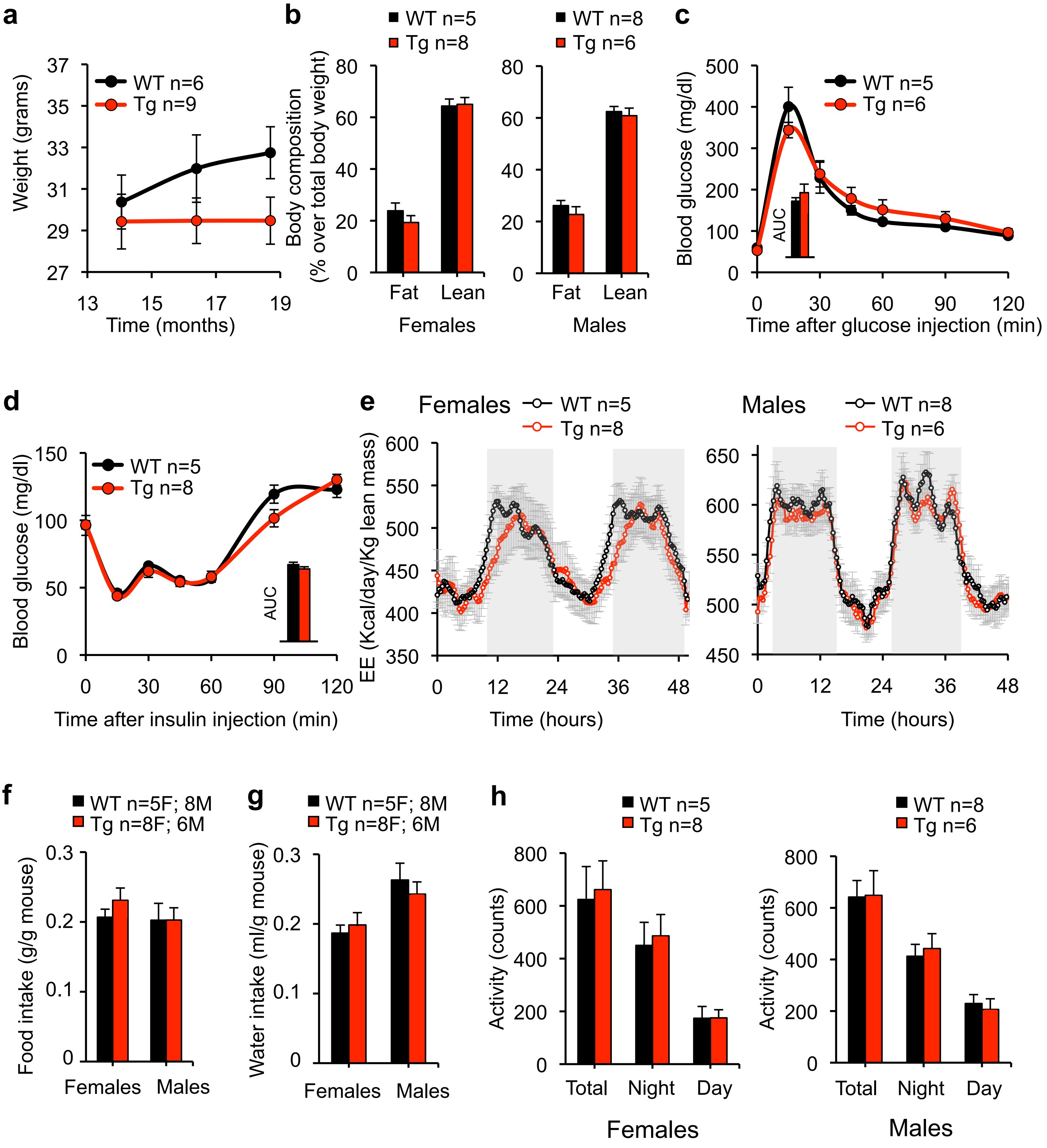

Supplementary Figure 3. Metabolic phenotype of G6PD-Tg mice. (**a**) Weight of female mice. (**b**) Body composition at 1 year of age. (**c**) GTT on 1 year-old females. Inset: area under the curve (AUC). (**d**) ITT on 1 year-old females. Inset: area under the curve (AUC). (**e-h**) Complete monitoring on 1 year-old animals, including (e) energy expenditure (EE); food (f) and water (g) intake, and activity (h). Values represent the mean ± the standard error of the mean (s.e.m.). Statistical significance was assessed using the two-tailed Student´s t test in all panels except panel (e), for which statistical significance was assessed using the ANCOVA test. No significant differences were observed.

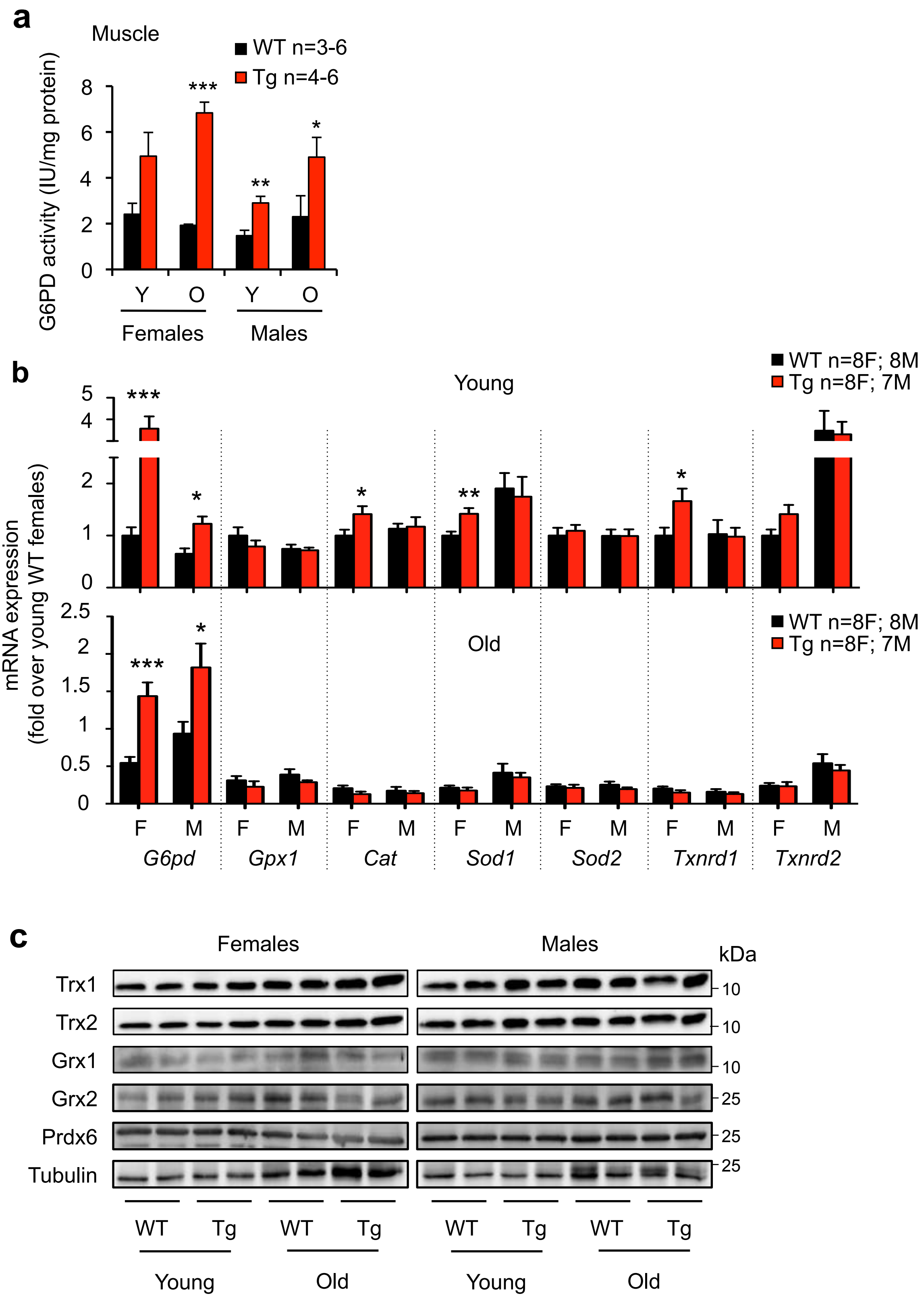

Supplementary Figure 4. Expression and activity of G6PD and other ROS detoxifying enzymes during age. (a) G6PD activity in gastrocnemius muscle from young (Y) or old (O) mice. **(b)** mRNA levels of the indicated genes in young (top panel) or old (bottom panel). **(c)** Immunoblots of the indicated proteins in liver samples. Values represent the mean ± the standard error of the mean (s.e.m.). Statistical significance was assessed using two-tailed Student´s t test: *, p<0.05. **, p<0.01. ***, p<0.001.

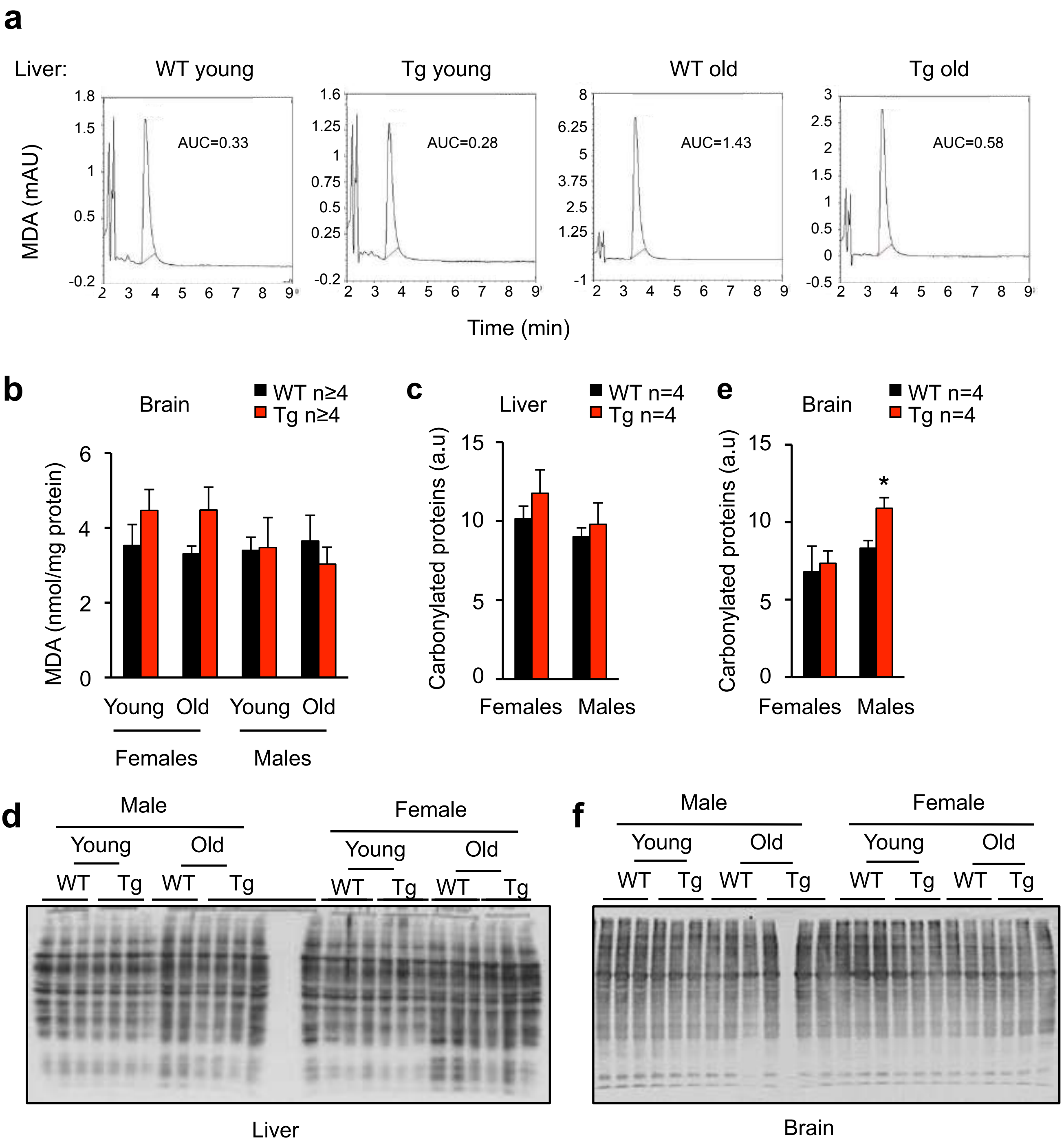

Supplementary Figure 5. Markers of ROS damage during age. (a) Representative chromatograms of MDA in liver samples from female mice. The AUC is later made relative to the protein concentration of the sample and to an MDA standard curve, to yield the final units (nmol/mg protein). **(b)** MDA levels in brain from the indicated mice. **(c-f)** Carbonylated proteins levels from liver (c, d) and brain (e, f) samples from old mice. Values represent the mean ± the standard error of the mean (s.e.m.). Statistical significance was assessed using the two-tailed Student´s t test: *, $p<0.05$.

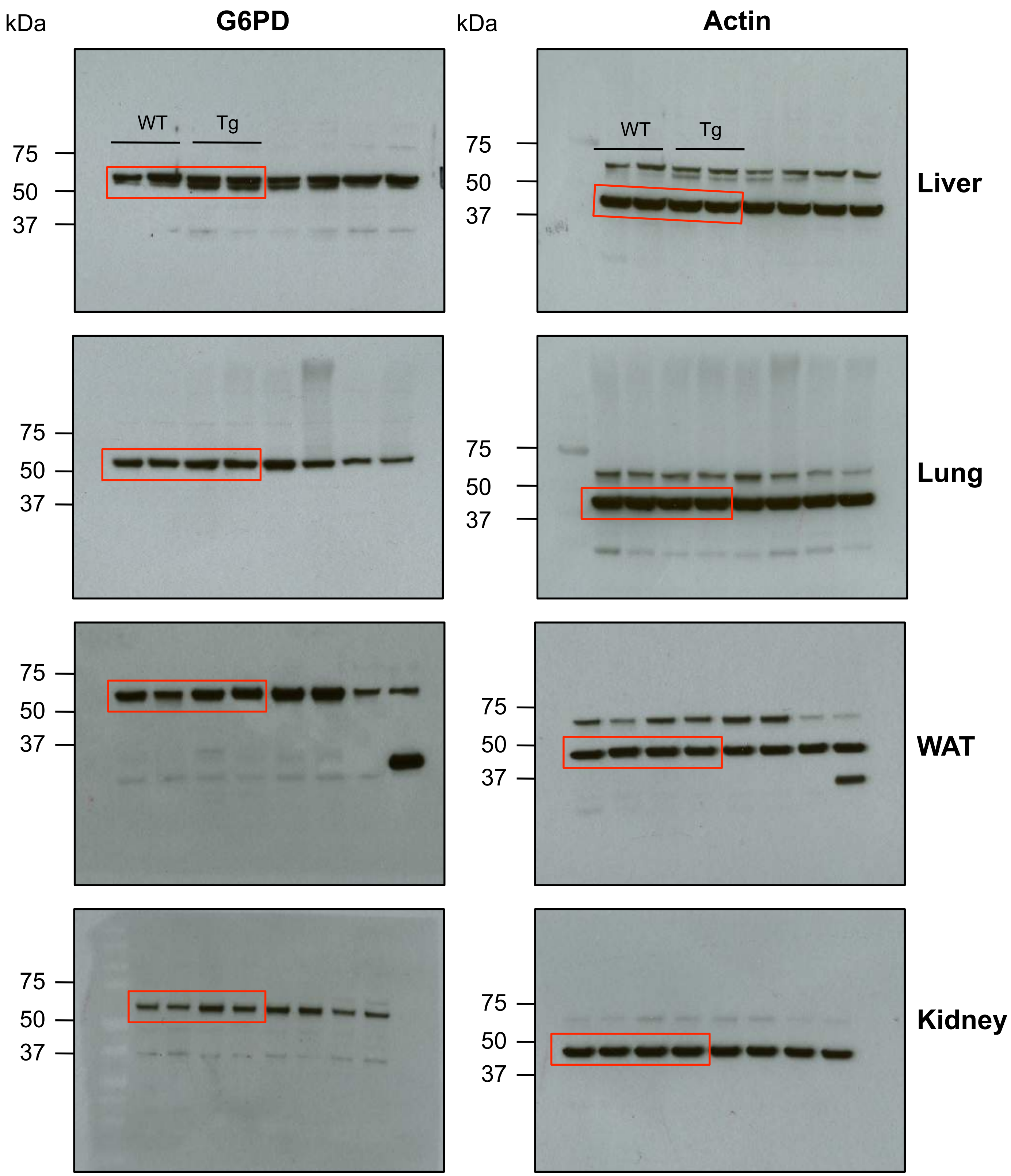

Supplementary Figure 6: Uncropped membranes for Figure 1b

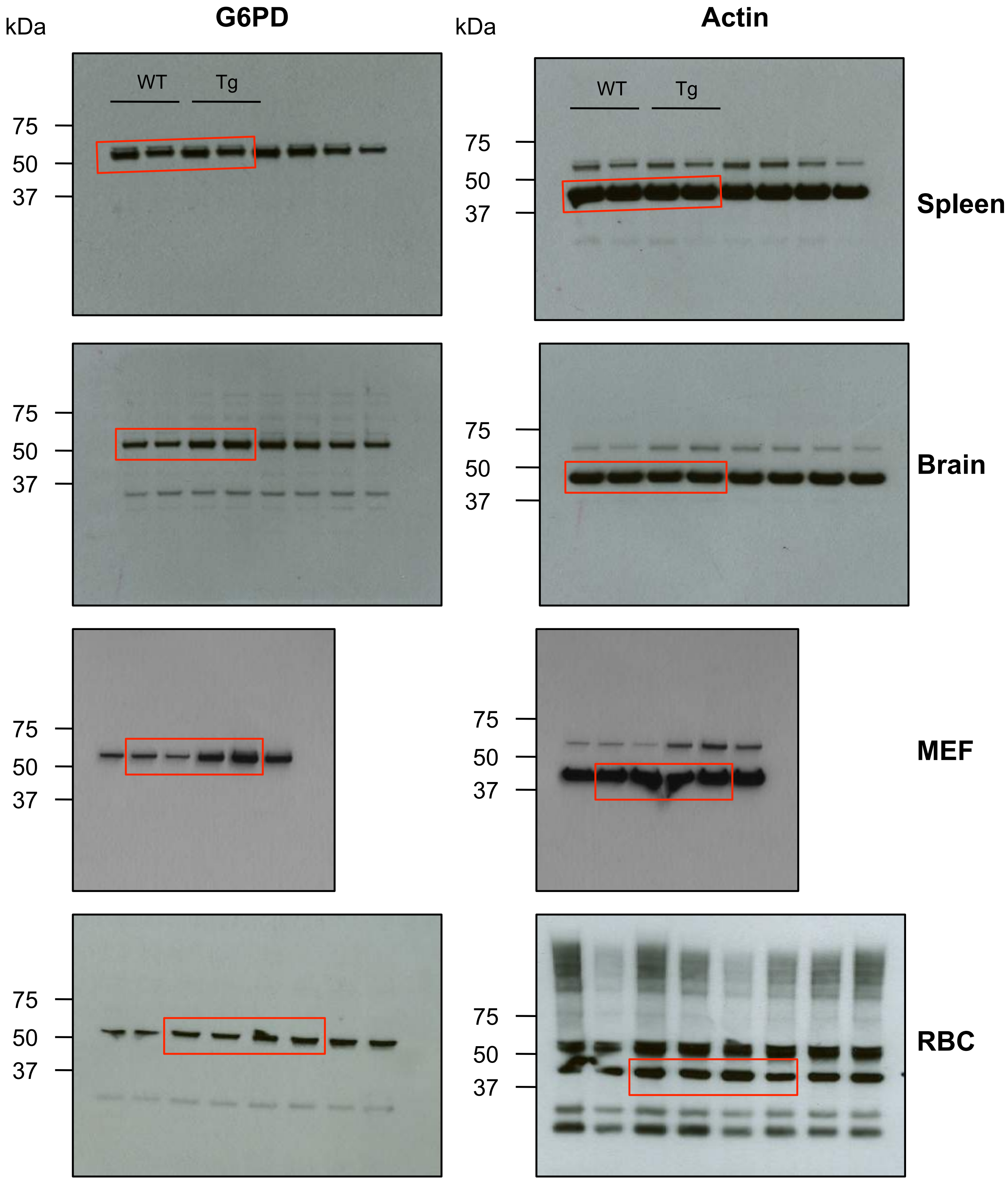

Supplementary Figure 6: Uncropped membranes for Figure 1b

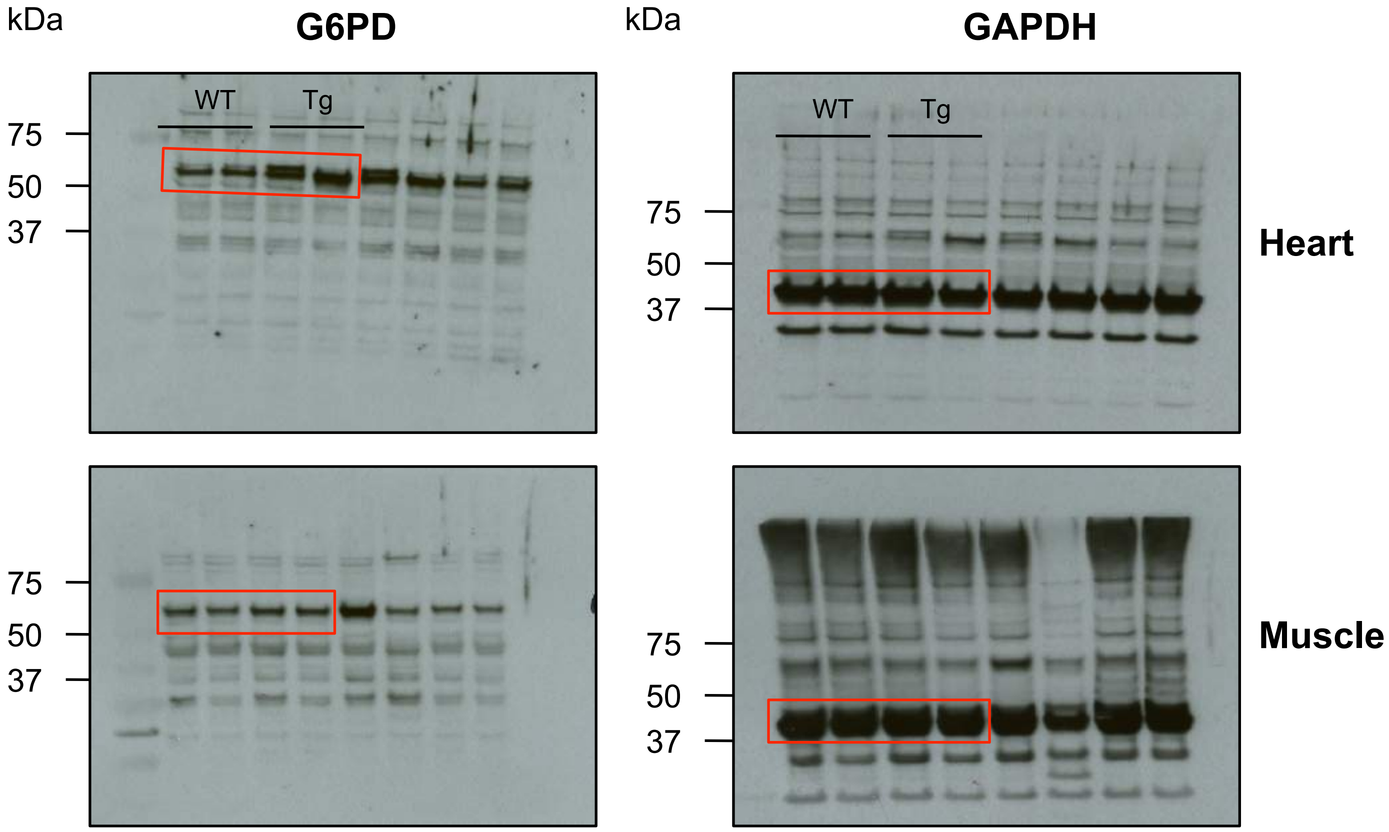

Supplementary Figure 6: Uncropped membranes for Figure 1b

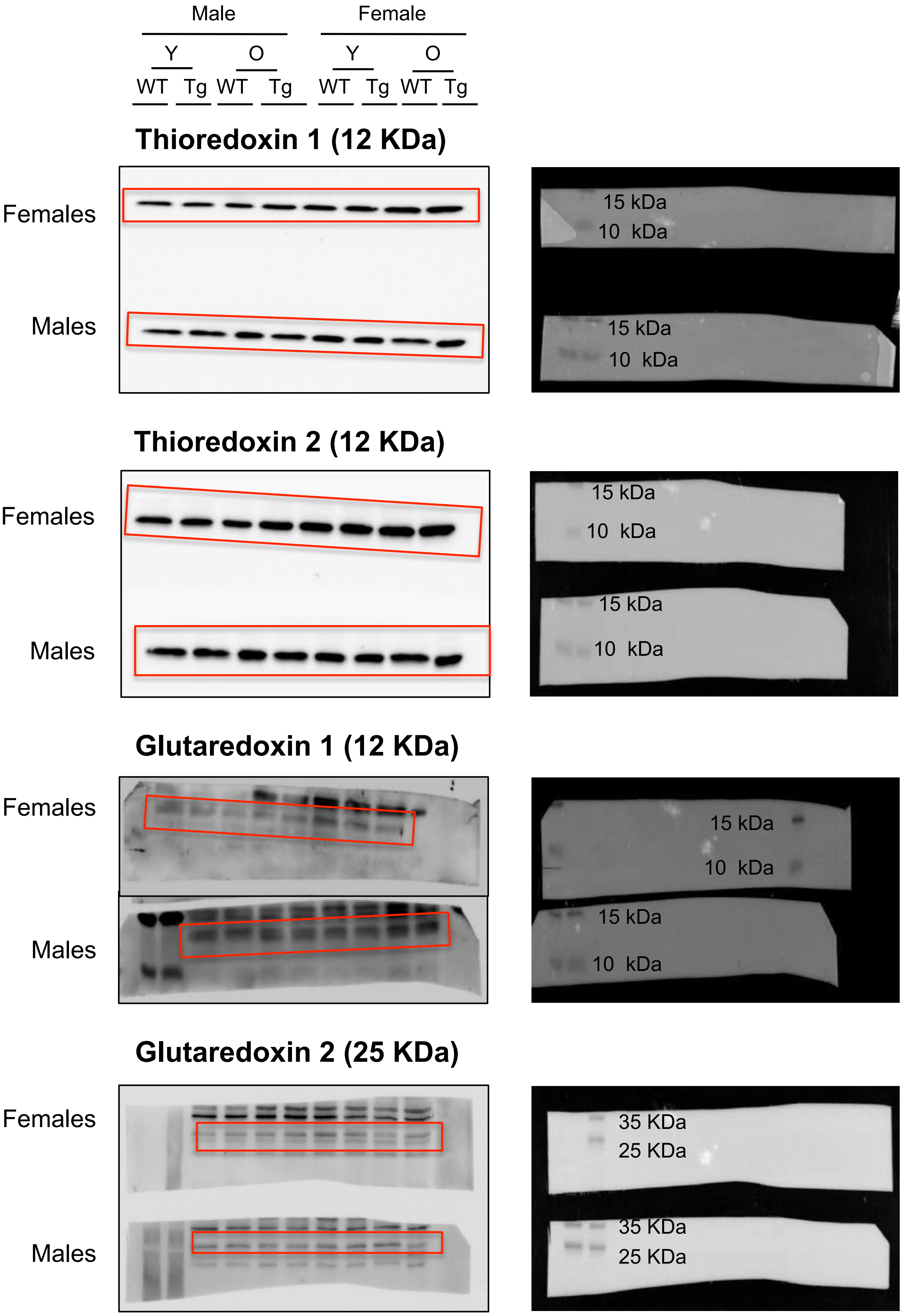

Supplementary Figure 7: Uncropped membranes for Supplementary Fig. 4c

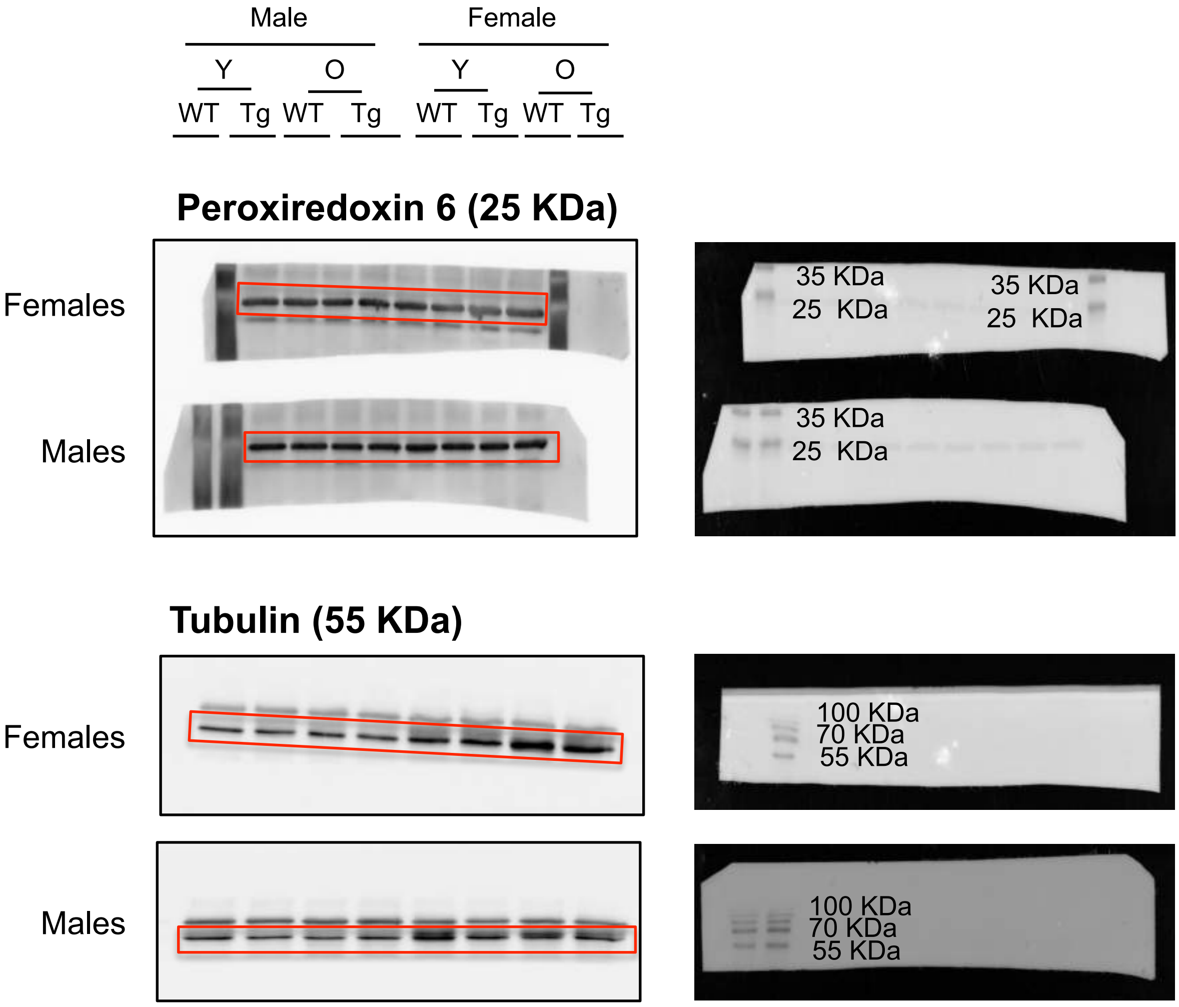

Supplementary Figure 7: Uncropped membranes for Supplementary Fig. 4c

Supplementary Table 1. Primers used for the measurement of mRNA levels by quantitative RT-PCR.

name	sequence
m*Actin*-F	5'-GGCACCACACCTTCTACAATG-3'
m*Actin*-R	5'-GTGGTGGTGAAGCTGTAGCC-3'
m*G6pd*-F	5'-CCGGAAACTGGCTGTGCGCT-3'
m*G6pd*-R	5'-CCAGGTCACCCGATGCACCC-3'
h*G6PD*-F	5'-GGAGGGCGACGACGACGAAG-3'
h*G6PD*-R	5'-TCGGGCAGAAGGCCATCCCG-3'
m*Gpx1*-F	5'-GTTTCCCGTGCAATCAGTTC-3'
m*Gpx1*-R	5'-CATTCCGCAGGAAGGTAAAG-3'
m*Cat*-F	5'-AGCGACCAGATGAAGCAGTG-3'
m*Cat*-R	5'-TCCGCTCTCTGTCAAAGTGTG-3'
m*Sod1*-F	5'- CCAGTGCAGGACCTCATTTT-3'
m*Sod1*-R	5'-TTGTTTCTCATGGACCACCA-3'
m*Sod2*-F	5'-GGCCAAGGGAGATGTTACAA-3'
m*Sod2*-R	5'-GCTTGATAGCCTCCAGCAAC-3'
m*Txnrd1*-F	5'-CAGCGAGGAGACCATAGAGG-3'
m*Txnrd1*-R	5'-GCACATTGGTCTGCTCTTCA-3'
m*Txnrd2*-F	5'-GCTTCTGGCAAGGAAGACAC-3'
m*Txnrd2*-R	5'-CCCTCAGCAACATCTCCAAT-3'
